# Biomimetic hybrid bioactive glasses gelatin composite rescues alveolar socket healing from diabetic impairment

**DOI:** 10.1186/s12903-026-08603-0

**Published:** 2026-05-26

**Authors:** Eman M. Salem, Asmaa M. Abd El-Aziz, Wafaa Yahia Alghonemy, Rania A. Hanafy

**Affiliations:** 1 Department of Oral Biology, Faculty of Dentistry, Pharos University in Alexandria, Sidi Gaber, P.O. Box 37, Alexandria, Egypt; 2 Fabrication Technology Research Department, Advanced Technology and New Materials Research Institute (ATNMRI), City of Scientific Research and Technological Applications (SRTA-City), Alexandria, Egypt; 3 Department of Basic Dental Sciences, Faculty of Dentistry, Zarqa University, Zarqa, Jordan; 4 Department of Oral Biology, Faculty of Dentistry, Tanta University, Tanta, Egypt; 5 Dental Materials Department, Faculty of Dentistry, Pharos University in Alexandria, Alexandria, Egypt

**Keywords:** Bioactive glass, Gelatin composite, Borosilicate glass, Alveolar socket healing, Diabetes mellitus, Bone regeneration

## Abstract

**Background:**

Diabetic individuals typically experience difficulties in healing their alveolar sockets due to impaired angiogenesis, persistent inflammation, and reduced osteoblastic activity. The development of biomimetic scaffolds that promote osteogenesis and restore the regenerative ability of diabetic bone remains a clinical necessity. This study aimed to evaluate the efficacy of novel gelatin-based hybrid composites incorporating silicate bioactive glasses (SBG) and borosilicate bioactive glasses (BBG) in facilitating alveolar bone regeneration in a diabetic rat model.

**Materials and methods:**

The sol–gel method was employed to synthesize SBG and BBG, which were subsequently incorporated into a five wt.% gelatin matrix to produce composite hydrogels designated as G-SBG and G-BBG. Five groups of 60 male albino rats were established: Group I (negative control), Group II (positive control), Group III (gelatin), Group IV (G–SBG), and Group V (G–BBG). Diabetes was induced, followed by the extraction of the bilateral lower first molars. Subsequently, the respective materials were implanted into the extraction sockets. After a period of recovery, mandibles underwent histological, scanning electron microscopy, and histomorphometric evaluations. Bone histomorphometry was analyzed using one-way ANOVA and Tukey's post hoc test (*p* < 0.05).

**Results:**

The G–BBG composite exhibited advanced structural and biological characteristics. It exhibited interconnected porosity, controlled swelling, and uniform ion distribution. Histologically, Group V had new bone formation, enlarged vascular marrow gaps, osteon development, and a lack of inflammatory infiltration. Statistical analysis throughout the two periods revealed that group V (G–BBG) demonstrated a significant difference (*p* < 0.05) when compared to groups II (+ ve control), III (gelatin), and IV (G–SBG), whereas no significant difference was seen between group V (G–BBG) and group I (-ve control).

**Conclusion:**

Incorporating BBG into a gelatin scaffold significantly enhanced the healing of alveolar bone in diabetic rats, restoring the bone structure to nearly normal levels. The gelatin/BBG hybrid composite serves as a promising biomimetic material for preserving the alveolar ridge and regenerating bone in patients with compromised healing capacity.

**Graphical Abstract:**

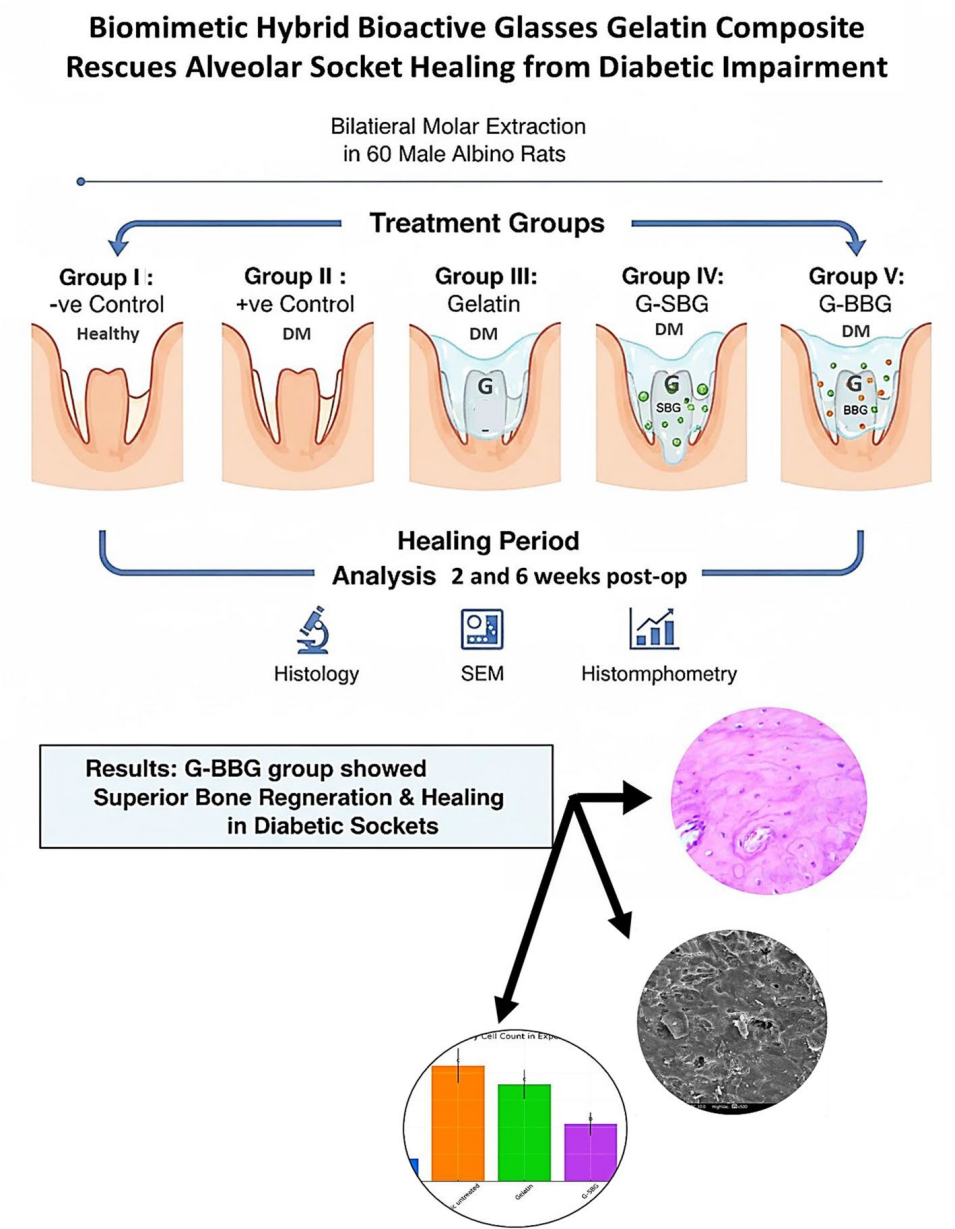

## Highlights

• Gelatin/borosilicate bioactive glass composite optimistically revived socket bone healing in diabetic rats.

• Gelatin/silicate bioactive glass composite promotes the healing of the diabetic extraction socket.

• Borosilicate bioactive glass enhanced the bone regenerative potential of gelatin in diabetic rats.

• The normal histological characteristics of the alveolar bone were repaired by a novel gelatin composite soon after tooth extraction in diabetic rats.

## Introduction

After a tooth is removed, the socket normally enters a self-directed healing phase, and in many cases, this occurs without the need for additional clinician intervention. This response depends on external biological signals, but sometimes it is not always strong enough to maintain ridge dimensions when substantial tissue loss is present. Healing is not a time-dependent event; some defects do not resolve predictably on their own, and the repair process is disrupted, particularly when the biological environment is compromised. Systemic conditions including diabetes mellitus (DM), osteoporosis, and states of immunosuppression as well as local infection or inadequate oral hygiene. The influences may delay new bone formation and alter tissue architecture. Consequently, relying exclusively on the patient’s own osteoprogenitor cells is often insufficient to fully restore the alveolar defect [1, 2].

Following tooth extraction, the alveolar ridge loses volume in both height and width, which can complicate the insertion of fixed prosthetics or implants. Clinicians commonly use grafting procedures such as autogenous bone or various substitute materials to limit this bone collapse and encourage new bone formation [3]. Autografts remain the benchmark because of their biological compatibility, yet their use is often constrained by donor-site morbidity, limited supply, and unpredictable resorption. These limitations have driven interest toward synthetic or natural biomaterials that can act as bioactive scaffolds not only to support but also stimulate tissue regeneration [3, 4].

Recent advances have shifted to the utilization of principles from engineering and life sciences to develop biomaterials that stimulate tissue and bone regeneration. They are considered a better option to eliminate donor site discomfort or morbidity that can occur in the case of an autograft. Gelatin is a biodegradable variant of collagen. Among naturally derived polymers, it has gained considerable interest because, as a component of the natural extracellular matrix, it can closely mimic the native tissue microenvironment. Alongside these advantages, it also incorporates well into composite scaffolds and is valued for its biocompatibility and its ability to support cell attachment and growth. Nonetheless, its mechanical properties are inferior to those of synthetic polymers. In this context, additional support, such as silicate-based bioactive glass (SBG) and borosilicate-based bioactive glass (BBG), is proposed [5].

Bioactive glasses (BGs), particularly those originating from silicate systems, exhibit remarkable capabilities in adhering to bone tissue and promoting osteogenesis and angiogenesis. Larry Hench introduced 45S5 Bioglass, composed of Na₂O–CaO–SiO₂–P₂O₅ oxides [5–7]. It is commercially referred to as "bioglass". One of the most important reactions in bioactive glasses is the so-called bioactivity mechanism, which involves the formation of the Hydroxycarbonate Apatite (HCA) layer [8]. This discovery revolutionized the approach to bone repair [9].

Bioactive glass is composed of quaternary inorganic oxides, Na_2_O–CaO–SiO_2_–P_2_O_5_.The immersion of bioactive glasses in biological fluid triggers their degradation. The release of ion products during degradation [10], especially Si and Ca, plays a pivotal role in its biological activity, as these ions can interact directly with surrounding cells and regulate osteoblast differentiation. [8, 9].

Over the years, numerous altered glass formulations have been developed by adding additional ions to enhance their biological and mechanical properties. Boron-doped bioactive glasses have compelling prospects as boron accelerates apatite formation, enhances angiogenesis, and promotes osteogenic differentiation. The use of boron enhances the glass network's reactivity, thereby accelerating healing by facilitating rapid transformation of the glass into the HCA layer upon contact with physiological fluids. Borosilicate bioactive glass (BBG) is particularly advantageous for dental and maxillofacial applications. It has also gained special attention as a dual agent for antibacterial properties and tissue regeneration, offering synergistic possibilities that enable dose reduction and cost savings [10–13].

Considering these advantages, the gelatin-bioactive glass composite is a biomimetic approach to creating hybrid scaffolds that integrate the biological affinity of natural polymers with the osteogenic properties of inorganic glasses. These composites can facilitate cellular proliferation, angiogenesis, and osteogenesis, even in cases of protracted recovery, such as in individuals with DM. This research aimed to construct and describe two novel gelatin-based hybrid scaffolds: gelatin/silicate bioactive glass (G–SBG) and gelatin/borosilicate bioactive glass (G–BBG). To the best of our knowledge, this is the first study to evaluate the synergistic effect of a gelatin/borosilicate bioactive glass (G–BBG) on the healing kinetics of extraction sockets in a diabetic rat model. While borosilicate glasses have shown promise in soft tissue repair, their application in the complex, pro-inflammatory environment of the diabetic alveolar bone encapsulated in a gelatin matrix for controlled delivery represents a novel therapeutic strategy.

## Materials and methods

### Ethical approval

All experimental procedures were executed in accordance with the guidelines established by the Research Ethics Committee, Faculty of Pharmacy, Pharos University, Alexandria, Egypt (Ethical Code: PUA-02–2024-09–29-3–268). Animal handling and care were conducted in accordance with the ARRIVE guidelines [14]. This study did not involve human participants; therefore, informed consent from adults or legal guardians of minors was not required.

### Materials

All chemicals used in the fabrication of scaffolds were sourced from Sigma-Aldrich (USA) and used as received, without further purification.

### Preparation of silicate and borosilicate bioactive glasses

Two types of bioactive glasses were prepared: silicate bioactive glass (SBG) with a composition of SiO₂:CaO: P₂O₅ (70:24:6 wt.%), and borosilicate bioactive glass (BBG) with a composition of SiO₂:CaO: P₂O₅:B₂O₃ (55:24:6:15 wt.%). Furthermore, for SBG, tetraethyl orthosilicate (TEOS, 35.4 mL) was used as the silica precursor, mixed with distilled water (21.6 mL), 2 M nitric acid (4.35 mL), and ethanol (100 mL). The mixture was stirred magnetically for 1 h at room temperature to ensure complete acid hydrolysis of TEOS according to the reaction:

$$\mathrm{S}\mathrm{i}{\left({\mathrm{O}\mathrm{C}}_{2}{\mathrm{H}}_{5}\right)}_{4}+{\mathrm{H}}_{2}\mathrm{O}\to 4\mathrm{S}\mathrm{i}\mathrm{O}\mathrm{H}+{4\mathrm{C}}_{2}{\mathrm{H}}_{5}\mathrm{O}\mathrm{H}$$

Triethyl phosphate (TEP, 1.7 mL) was then added as the phosphorus source, followed by calcium nitrate tetrahydrate (Ca (NO₃)₂·4H₂O, 11.214 g) as the calcium source, both of which were stirred for 30 min. For BBG, TEOS (27.68 mL) was mixed with distilled water (16.88 mL), 2 M nitric acid (3.4 mL), and ethanol (100 mL), and stirred for 1 h to facilitate hydrolysis. TEP (1.7 mL) and Ca (NO₃)₂·4H₂O (11.214 g) were added sequentially with 30-min stirring intervals. Finally, boric acid (H₃BO₃, 6.9 g) was added as the boron source and stirred for an additional 30 min [15–17].

In both preparations, 30 mL of ammonium solution (2 M) was added dropwise while vigorously stirring to initiate gelation, which occurred within 2–3 min. The gels were dried at 80 °C for 48 h, followed by sintering at 700 °C for two h (heating rate: 3 °C/min) to obtain fine bioactive glass powders.

### Preparation of gelatin-based hydrogel composites

A 5 wt.% gelatin solution was prepared in deionized water at 40 °C. Bioactive glass powders (SBG or BBG) were added to the gelatin solution at a concentration of 50 mg mL⁻^1^ and stirred for two h to obtain homogeneous dispersions. The mixtures were crosslinked using 5 µL mL⁻^1^ glutaraldehyde (GTA) and stirred for an additional hour at room temperature. The prepared hydrogels were then lyophilized and allowed to swell in phosphate-buffered saline (PBS, pH 7.4, 37 °C) for 5–60 min. The swelling ratio was calculated using the equation:

$$Swelling\;ratio\;(\%)=\frac{{W}_{s}-{W}_{0}}{{W}_{0}}\times 100$$

where *W₀* is the dry weight, and *Wₛ* is the swollen weight. Values were expressed as mean ± SD (*n* = 3).

### Characterization of the prepared scaffolds

The physicochemical properties of the prepared materials were assessed using the instruments in Table 1

**Table 1:** The physicochemical properties of the prepared materials were assessed using the following instruments

Instrument	Model	Data	Conditions
Fourier-Transform Infrared Spectrometer (FTIR)	Bruker VERTEX 70	FTIR spectra	400–4000 cm⁻^1^
X-Ray Diffractometer (XRD)	Shimadzu LabX 6100 (Kyoto, Japan)	XRD patterns	Cu-Kα (λ = 1 Å); 40 kV, 30 mA; 2θ = 10°–80°, step = 0.02°, 0.6 s/time, 25 °C
Scanning Electron Microscope (SEM)	JSM-lT200, JEOL Ltd	SEM images	Sputter-coated (JEOL JFC-1100E)
High-Resolution Transmission Electron Microscope (HR-TEM)	JEOL JSM-1400 plus	TEM images	Imaging mode
Energy-Dispersive X-ray Spectroscopy (EDX)	JSM-lT200, JEOL Ltd	EDX spectra	20 kV acceleration voltage, WD 10 mm, live time 30 s, high vacuum mode

### Sample size calculation

The sample size was determined based on a previous study investigating the effects of boron and fish oil on extraction socket healing in rats [20]. The minimum sample size was 12 rats per group, yielding a total of 60 specimens (12 × 5 = 60 animals). This was predicted based on a standardized effect size of 0.814 and a power of 80% (β = 0.20) at an α level of 0.05. To maintain uniform group sizes, any samples lost during processing were substituted [18]. To eliminate any ambiguity, a computer-generated sequence of random integers was used to determine treatment status. The individual and all study team members were oblivious to the treatment allocation.

### Group assignment and animal preparation

We acquired 60 healthy male albino rats (8–10 weeks old, weighing 200–250 g) with intact dentition from the Pharos University animal facility. All animals were obtained from the Animal Research Facility at the Faculty of Pharmacy, Pharos University, Alexandria, Egypt. Two weeks before the experiment, the rats were selected and examined to ensure they were free of any general or dental issues. Throughout the study, the animals were provided with sustenance and hydration. The subjects were maintained in a controlled setting with regulated illumination and temperature, in accordance with the ARRIVE guidelines and institutional animal welfare standards [14, 19].

### Induction of DM

Initially, all rats underwent a 12-h fasting period to standardize their baseline metabolic parameters. Type 1 DM was induced by a single intraperitoneal administration of streptozotocin (STZ) at a dosage of 50 mg/kg body weight, freshly reconstituted in 0.1 M citrate buffer (Sigma-Aldrich, USA; pH 4.5), just before injection. DM was confirmed 72 h post-injection by fasting blood glucose levels > 250 mg/dL (measured via glucometer, i-SENS, Inc., South Korea) [20]. Rats were classified as diabetic when fasting glucose levels exceeded 250 mg/dL, in accordance with previously published criteria [21]. After confirmation of DM at 72 h post-STZ injection, animals were monitored throughout the experiment by serial blood glucose measurements. Rats that did not maintain hyperglycemia above 250 mg/dL were excluded from further analysis—Table 2.

**Table 2:** Fasting Blood Glucose Levels (mg/dL)

Group	Baseline BGL	post-STZ	End of Study BGL
Control rats	91 ± 7	96 ± 7	99 ± 6
Diabetic rats	100 ± 8	289 ± 25*	301 ± 33*

^*^Significantly different from the Control group (*p* < 0.05). Diabetes was confirmed when BGL > 250 mg/dL

### Tooth extraction technique

**Fig. 1:**
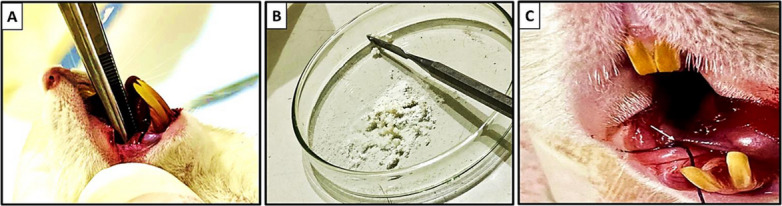
**A** Tooth extraction technique, **B** Preparation of Gelatin-Based Hydrogel Composites. **C** Alveolar sockets suturing with 4–0 black silk

Spiramycin (7 mg/kg; Pharaonia Pharmaceuticals, Egypt) and metronidazole (12 mg/kg; Sanofi Aventis, Egypt) were administered orally to all rats three days before tooth extraction to avert postoperative infections. Diabetic rats underwent fasting for one to two hours before the surgical procedure. To mitigate procedural stress and reduce the risk of anesthesia-induced hypoglycemia, free access to water was granted. To diminish salivary secretions during tooth extraction, animals received an intramuscular injection of atropine sulfate at a dosage of 0.4 mL/kg. An intramuscular combination of 2% xylazine (Adwia, 10th of Ramadan City, Egypt) and 10% ketamine hydrochloride (Ketamine Alfasan 10%, Woerden, The Netherlands) was administered at doses of 0.2 mL/kg and 0.5 mL/kg body weight, respectively, to induce general anesthesia [22, 23]. Rats from every group underwent bilateral mandibular first molar tooth extraction, according to Moghadam et al. [24]. The surgical site was initially disinfected with an iodine swab. Subsequently, each tooth was luxated utilizing surgical elevators by gently angling it for one second in both the buccal and lingual directions. This was performed ten times to loosen the teeth. The lower remaining root extraction forceps were thereafter employed to facilitate the effortless removal of the tooth (Fig. 1-A).

Then the rats were randomly assigned to five groups (*n* = 12 per group):

- Group I (-ve Control): Healthy, non-diabetic rats
- Group II (+ ve Control): Diabetic rats with extraction sockets left untreated
- Group III (Gelatin): Diabetic rats with extraction sockets filled with gelatin
- Group IV (G–SBG): Diabetic rats with sockets filled with gelatin/silicate bioactive glass composite
- Group V (G–BBG): Diabetic rats with sockets filled with gelatin/borosilicate bioactive glass composite

Following the implantation of materials in Groups III, IV, and V, the sockets were sutured with 4–0 black silk thread (Fig. 1 B and C). Postoperative animals were offered antibacterial and analgesic therapy: ampicillin (25 mg/kg, every 8 h for 5 days) and diclofenac (Cataflam, Novartis, Egypt) intramuscularly every 8 h for 2 days. [25].

### Animal euthanization and histological examination

After two and six weeks post-extraction of the mandibular first molar, six rats from each group were euthanized. Euthanasia Procedure: At the designated experimental endpoints (2 and 6 weeks), animals were humanely euthanized under deep anesthesia. General anesthesia was induced using an intraperitoneal injection of ketamine (90 mg/kg) and xylazine (10 mg/kg). The absence of pedal and corneal reflexes confirmed adequate depth of anesthesia. Subsequently, euthanasia was performed by cervical dislocation, following the AVMA Guidelines for the Euthanasia of Animals (2020). This method was selected because it ensures rapid loss of consciousness and is recommended for small laboratory rodents.

Subsequently, the rats were euthanized, and their mandibles were dissected and promptly fixed in 10% neutral-buffered formalin for 48 h. Samples were subsequently decalcified in a 10% ethylenediaminetetraacetic acid (EDTA) solution at ambient temperature, followed by standard dehydration in a series of increasing alcohol concentrations, clarification in xylene, and embedding in paraffin.

Serial sagittal sections measuring 4–5 μm in thickness were cut with a rotary microtome and affixed to glass slides. Sections were stained with hematoxylin and eosin (H&E) and Masson's trichrome stain for comprehensive tissue assessment. Slides were analyzed using a light microscope (LM). Representative photomicrographs were obtained utilizing a digital camera system connected to the microscope (Leica ICC50 HD), with images of distinctive regions acquired and annotated [26]. All histological evaluations were conducted by a seasoned oral biologist who was unaware of the experimental groups.

### Histomorphometric analysis

We conducted a quantitative histomorphometric analysis to examine the growth of fresh bone and the tissue response in the extraction sockets. We utilized ImageJ software (National Institutes of Health, Bethesda, MD, USA) to analyze the LM micrographs. Five unique fields per specimen were randomly selected at × 400 magnification. The subsequent parameters were evaluated [27].

- New bone formation (%): the proportion of the socket area occupied by newly formed bone.
- Marrow space area (%): the proportion of the socket area constituted by marrow spaces.
- Inflammatory cell count: the mean quantity of inflammatory cells observed in a high-power field.

A calibrated examiner, unaware of the subjects' group assignments, conducted all measurements to mitigate observer bias. We calculated the mean values for each specimen and employed them for statistical comparison.

### Statistical analysis

Quantitative histomorphometric data were entered into IBM SPSS Statistics version 20.0 (Armonk, NY: IBM Corp.). We employed one-way ANOVA and the Tukey post hoc test to identify differences among the groups. The threshold for statistical significance was established at *p* < 0.05 [28].

## Results

### Clinical observations

Throughout the experiment, all animals exhibited typical activity and feeding behaviors, with no indications of pain or infection at the extraction sites. No complications or fatalities occurred post-surgery, and all the animals were euthanized promptly. Healing progressed effectively across all experimental groups.

### Structural characterization of the prepared hydrogel composites

#### Morphology

**Fig. 2:**
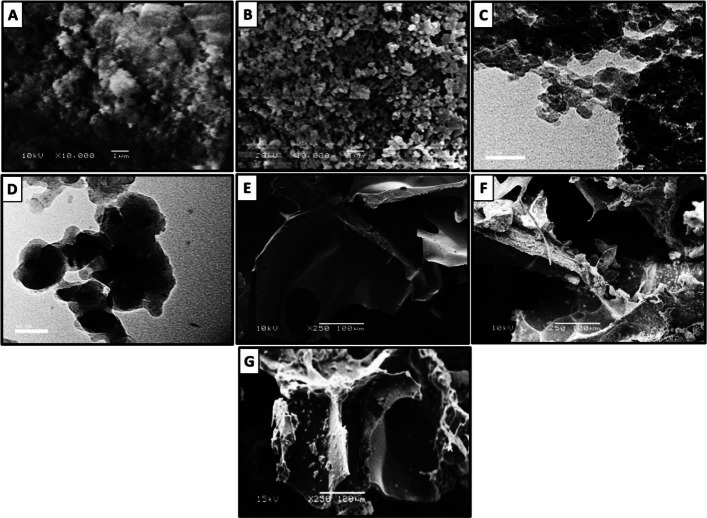
SEM and TEM micrographs of bioactive glass particles and composite hydrogels. **A**, **B** SEM of SBG and BBG particles (× 10,000); **C**, **D** TEM images of SBG and BBG (50 nm scale); **E**–**G** SEM of freeze-dried gelatin **G**, G–SBG, and G–BBG composites, respectively

The morphology of the obtained bioactive glass powders is illustrated in Fig. 2 (A–D). Scanning electron microscopy (SEM) revealed that both silicate (SBG) and borosilicate (BBG) particles were irregular in shape, non-spherical, and appeared as compact aggregates with sharp edges. These tertiary particles formed during gelation through the aggregation of secondary particles, which coalesced during sintering at 700 °C, producing a porous structure with interstitial voids. This nanoscale porosity contributes to the high surface area and reactivity of the glass particles. Transmission electron microscopy (TEM) images confirmed the nano-structural features of the synthesized glasses, with average particle sizes of approximately 111 ± 38 nm for SBG and 165 ± 31 nm for BBG. The interstitial voids visible between tertiary aggregates correspond to nanopores that facilitate ion exchange and bioactivity. Following lyophilization, the gelatin-based hydrogel composites appeared yellowish and highly porous. SEM micrographs (Figs. 2E–G) revealed that all composite scaffolds displayed a well-connected, three-dimensional porous architecture with average pore diameters ranging from 110 to 290 µm. Such an interconnected porous network is crucial for nutrient diffusion, cell infiltration, and angiogenesis. Gelatin served as a structural matrix, while the bioactive glass particles were embedded within the pore walls, providing mechanical stability and osteoconductive surface features. Notably, the incorporation of SBG particles (Group IV) increased the average pore size compared with gelatin alone, whereas BBG particles (Group V) produced a slightly denser structure with smaller pores. This difference likely resulted from particle agglomeration within the gelatin matrix (Fig. 2F–G).

### FT-IR Analysis

**Fig. 3:**
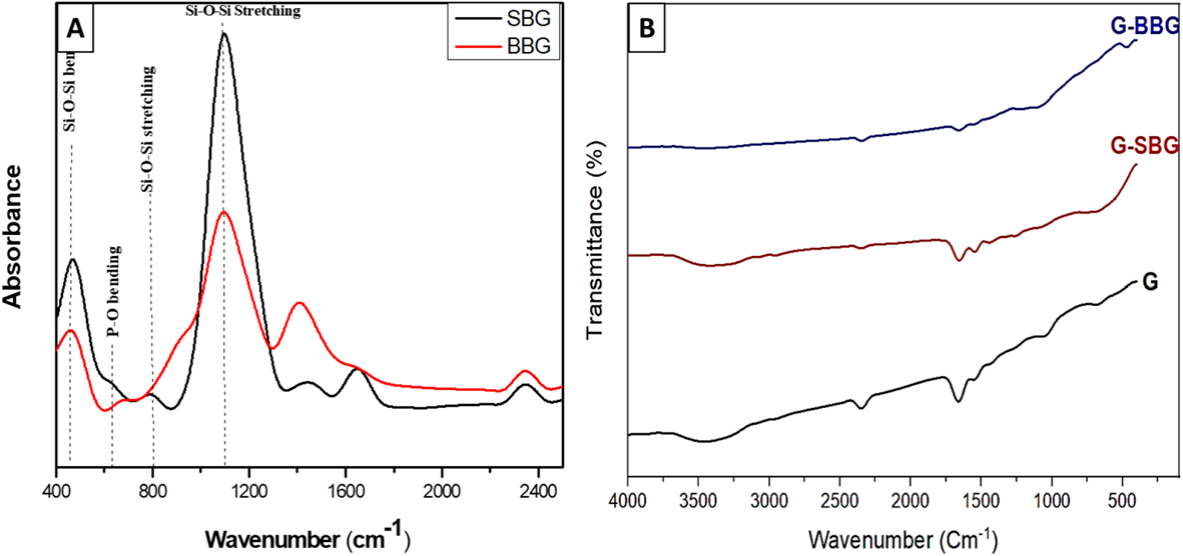
FT-IR spectra of: **A** Silicate and borosilicate bioactive glass powders; **B** Gelatin-based hydrogel composites (G, G–SBG, and G–BBG)

FT-IR spectra of SBG and BBG (Fig. 3A) exhibited broad absorption bands between 500–1076 cm⁻^1^ corresponding to Si–O–Si and B–O stretching vibrations of tetrahedral [BO₄] units. The peak at 812 cm⁻^1^ indicated Si–O–Ca (non-bridging oxygen), whereas a weaker band at 750 cm⁻^1^ corresponded to O–P–O bending vibrations. The shoulder around 600 cm⁻^1^, attributed to P–O bonds, diminished as the boron content increased, indicating network modification. Peaks at 1458 cm⁻^1^ (shifting to 1413 cm⁻^1^ with increasing B₂O₃) and the additional bands at 703 cm⁻^1^ and 1422 cm⁻^1^ confirmed the presence of BO₃ groups, demonstrating partial substitution of SiO₄^4^⁻ units by borate species in the glass structure. [1, 2, 29]. FT-IR spectra of the gelatin-based hydrogel composites (Fig. 3B) revealed characteristic amide peaks of gelatin at 3452 cm⁻^1^ (O–H stretching and amide A), 1649 cm⁻^1^ (amide I), 1533 cm⁻^1^ (amide II), and 1252 cm⁻^1^ (amide III). The band at 1036 cm⁻^1^ corresponded to the C–O stretching of glycerol residues. In the composite groups (G–SBG and G–BBG), minor peak shifts and intensity changes confirmed physical interactions and hydrogen bonding between gelatin and the incorporated bioactive glass particles.

### Swelling behavior

**Fig. 4:**
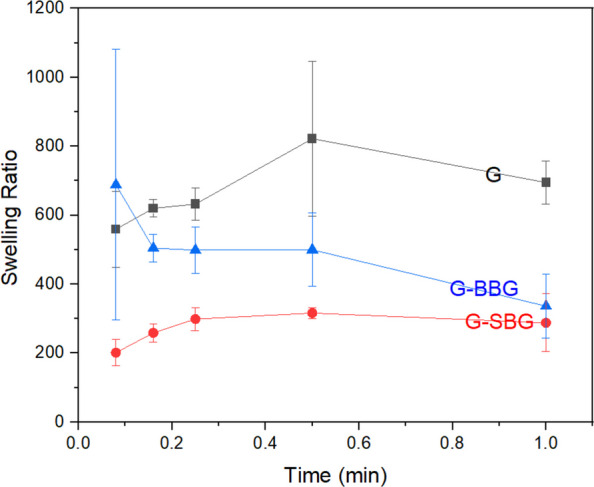
Swelling behavior (water uptake %) of gelatin (G), gelatin/silicate bioactive glass (G–SBG), and gelatin/borosilicate bioactive glass (G–BBG) scaffolds at various time intervals (mean ± SD, *n* = 3)

The swelling capacity of the hydrogel scaffolds is presented in Fig. 4. All formulations demonstrated rapid water uptake during the first 60 min, followed by a period of stabilization. Pure gelatin scaffolds exhibited the highest swelling ratio (694 ± 62%). In comparison, composites containing bioactive glass particles exhibited significantly lower swelling ratios, reduced by approximately 58.5% and 51.6% for the G–SBG and G–BBG scaffolds, respectively. The decrease in swelling capacity is attributed to increased crosslinking density and the filler effect of glass nanoparticles, which produce a denser polymer network with smaller pores. Controlled swelling under physiological conditions is crucial for preserving scaffold integrity and maintaining a stable environment for cellular infiltration and mineral deposition.

### Histological evaluation

#### A- Hematoxylin and eosin (H&E) stain:

**Fig. 5:**
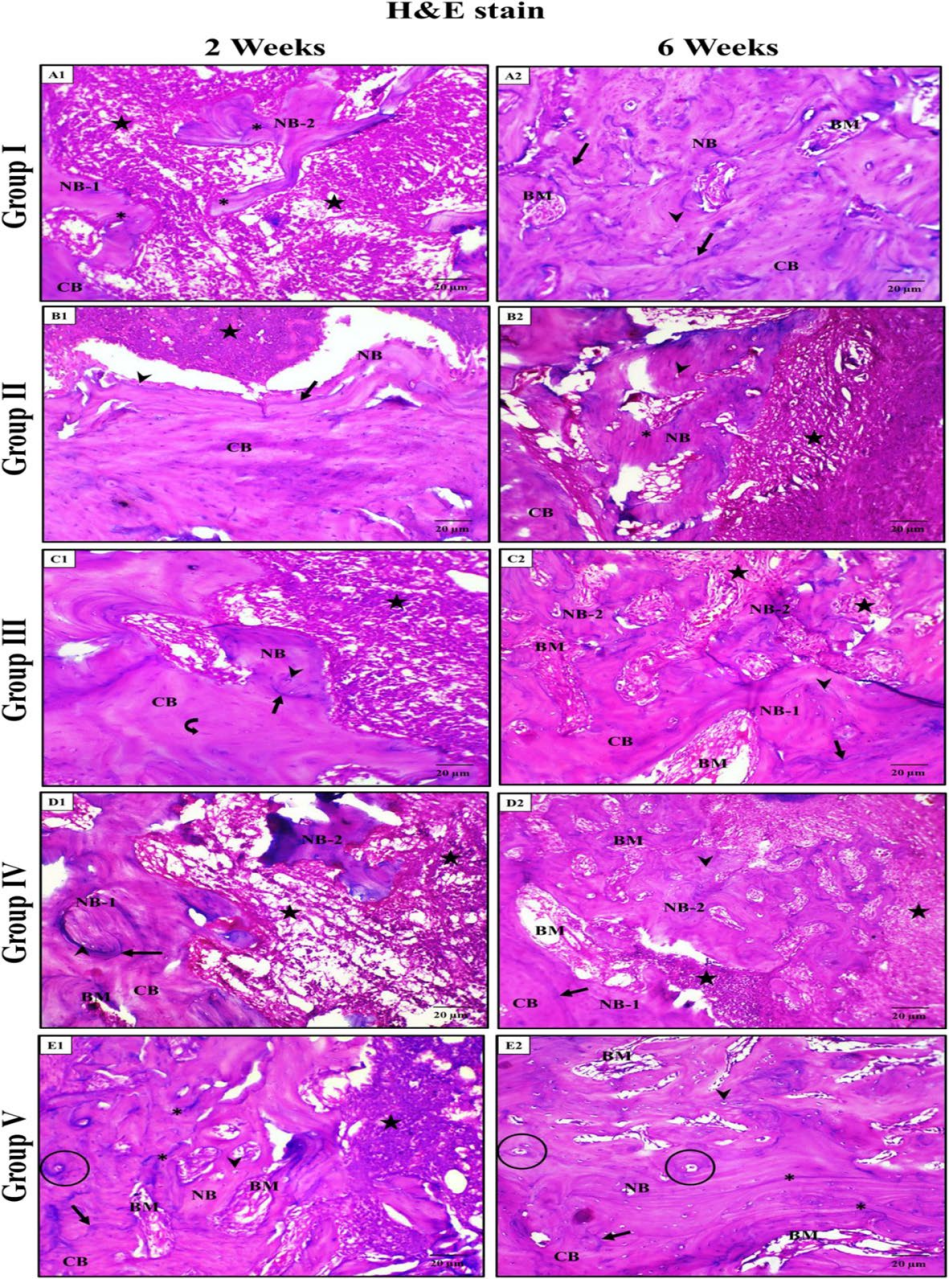
LM micrographs of H & E-stained sections for the extraction socket in all groups. (A1 and A2) group I (-ve control) at 2 and 6 weeks, respectively. A1, showing dense fibrous granulation tissue (stars) that plugs the socket area, mixed with the newly formed bone (NB-1 and NB-2), containing dense bone resting lines (*) near the cortical bone (CB) of the socket wall. A2, showing newly formed bone (NB) matrix filling the extraction socket, contains many large osteocytes (arrowhead), and many vascular bone marrow (BM) spaces, and dense bone resting lines (arrows), which indicate active bone formation areas, which differ from the mature CB of the socket wall. (B1 and B2) group II (+ ve control) at 2 and 6 weeks, respectively. B1, the extraction socket is filled with dense granulation tissue (stars) and a small area of NB, which contains prominent, large-sized osteocytes (arrow heads). It is separated from the CB of the socket wall with a dense bone resting line (arrow). B2, the socket is filled with dense fibrous granulation tissue (stars) and thin bone trabecula of NB, which contains dense bone resting lines (*) and many prominent, large-sized osteocytes (arrow heads). (C1 and C2) group III (Gelatin) at 2 and 6 weeks, respectively. C1, the extraction socket is filled with dense granulation tissue (star) and a small area of NB separated from the CB of the socket wall with a thick bone resting line (arrow), and it also contains prominent, large-sized osteocytes (arrowhead). C2, the socket area is filled with dense fibrous granulation tissue (stars) mixed with thick bone trabeculae of NB-2 and NB-1 separated by BM spaces, also contains many osteocytes (arrowheads), and is lined with a densely stained line (arrow) demarcating its boundary with CB of the socket wall. (D1 and D2) group IV (G–SBG) at 2 and 6 weeks, respectively. D1, the socket area is filled with dense fibrous granulation tissue (stars) mixed with a small free mass of bone NB-2 and another NB-1, which contains many osteocytes (arrowheads) and is lined with a densely stained line (arrow) demarcating its boundary with CB of the socket wall. D2, the socket is filled with dense fibrous granulation tissue (stars) and thin bone trabeculae of NB-2, which contain many prominent, large-sized osteocytes (arrowhead), and large BM spaces. The remodeling line (arrow) demarcates the boundary between the NB-1 and the CB of the socket wall. (E1 and E2) group V (G–BBG) at 2 and 6 weeks, respectively. E1, the socket is filled with dense fibrous granulation tissue (stars) and thick bone trabeculae of NB, which contains many large-sized osteocytes (arrowhead), bone resting lines (*), and many BM spaces. The circled area indicates osteon formation. The densely stained line (arrow) lines the NB from the native CB of the socket wall. E2, the NB bone matrix filling the extraction socket contains many large osteocytes (arrowhead), and vascular BM spaces, which differ from the mature CB of the socket wall. A dense bone resting lines (*), which indicate active bone formation areas. The circled areas indicate osteon formation. The densely stained line (arrow) lines the NB from the native CB of the socket wall. (H& E stain X 400)

*Group I (-ve control):* After two weeks, it showed dense fibrous granulation tissue that plugged the socket area, mixed with the newly formed bone, containing dense bone resting lines near the cortical bone of the socket wall. Then, at six weeks, it depicted a newly formed bone matrix filling the extraction socket, which contained many large osteocytes and numerous vascular bone marrow spaces, as well as dense bone resting lines indicating active bone formation areas, which differed from the mature bony wall of the socket (Fig. 5- A1 and A2).

*Group II (*+ *ve control):* After two weeks, the extraction socket appeared filled with dense granulation tissue and a small area of newly formed bone, which contained prominent, large-sized osteocytes. The newly formed bone is depicted as separated from the socket wall, with a dense line of bone resting. After six weeks, the socket was filled with dense fibrous granulation tissue and thin bone trabeculae of newly formed bone, which contained dense bone resting lines and many prominent, large-sized osteocytes (Fig. 5-B1 and B2).

*Group III (Gelatin):* After two weeks, the extraction socket appeared filled with dense granulation tissue and a small area of new bone separated from the native socket wall with a thick bone resting line, and it also contained prominent, large-sized osteocytes. After six weeks, the socket area was filled with dense fibrous granulation tissue mixed with thick bone trabeculae of newly formed bone separated by bone marrow spaces, also containing many osteocytes. It was lined with a densely stained line demarcating its boundary with the native socket wall (Fig. 5-C1 and C2).

*Group IV (G–SBG):* After two weeks, the socket area appeared filled with dense fibrous granulation tissue mixed with a small free mass of newly formed woven bone, which contained many osteocytes and was lined with a densely stained line demarcating its boundary with the socket wall. After six weeks, the socket was filled with dense fibrous granulation tissue and thin bone trabeculae of new bone, which contained many prominent, large-sized osteocytes, and large bone marrow spaces. The remodeling line demarcated the boundary between the new bone and the socket wall (Fig. 5-D1 and D2).

*Group V (G–BBG):* After two weeks, the socket was filled with dense fibrous granulation tissue and thick bone trabeculae of newly formed bone, which contained many large-sized osteocytes, bone resting lines, and many bone marrow spaces. Additionally, osteon formation was primarily observed during this period. The densely stained line appeared to line the new bone from the native bone of the socket wall. After six weeks, the new bone matrix filling the extraction socket contained many large osteocytes and vascular bone marrow spaces, which differ from the mature compact bone of the socket wall. A dense bone resting line and osteon formation, which indicated active bone formation areas, were depicted. In addition, the densely stained line that lined the newly formed bone from the native bone of the socket wall (Fig. 5-E1 and E2).

### B- Masson's trichrome stain

**Fig. 6:**
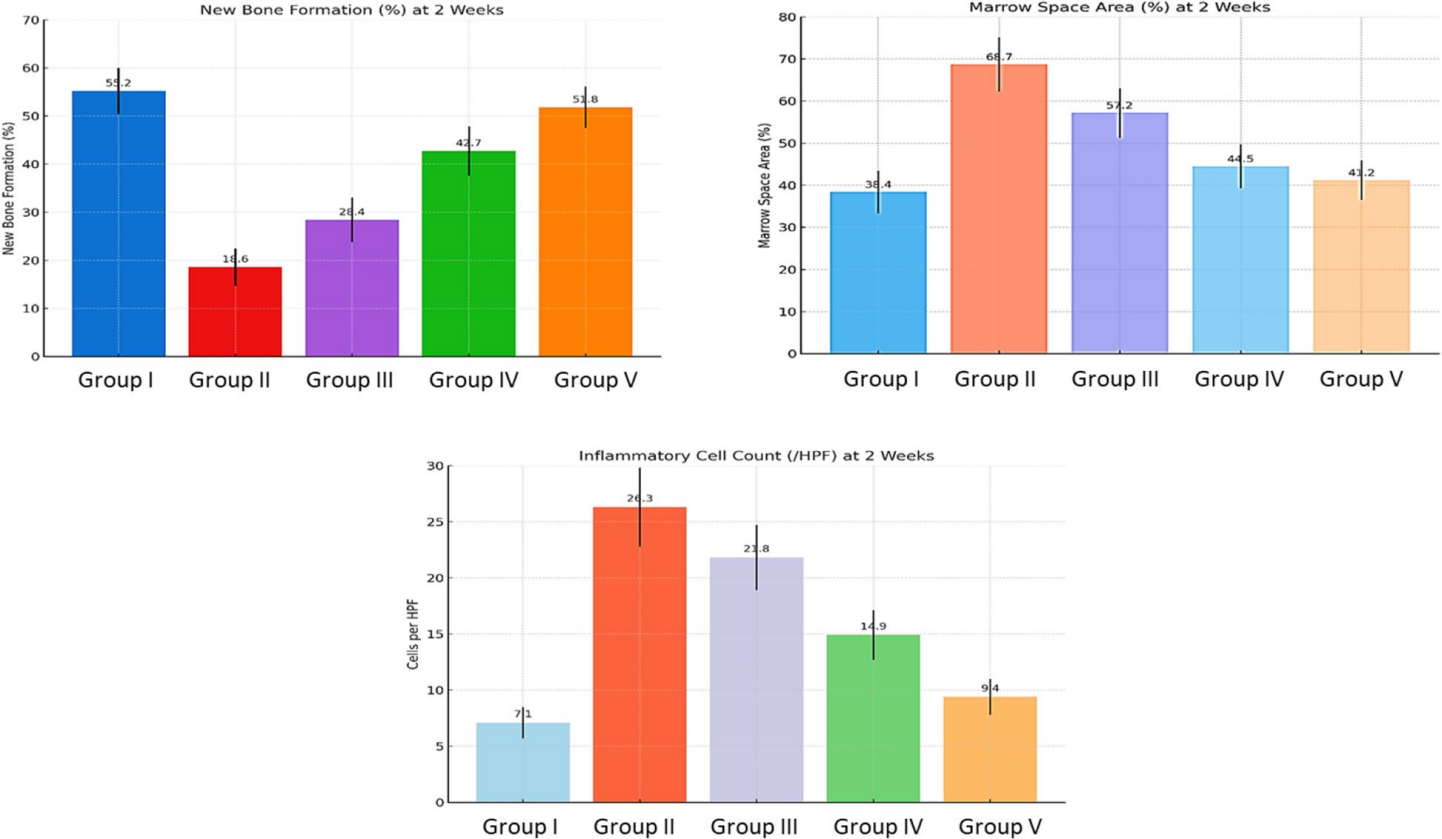
LM micrographs of Masson’s trichrome-stained sections for the extraction socket in all groups. (A1 and A2) group I (-ve control) at 2 and 6 weeks, respectively. A1, showing dense fibrous granulation tissue (stars) that fills the socket area, mixed with the newly formed bone (NB-1 and NB-2), containing dense bone resting lines (arrow). A2, showing newly formed bone (NB) matrix filling the extraction socket, contains many large osteocytes (curved arrow), and many vascular bone marrow (BM) spaces, and multiple dense bone resting lines (arrows), which indicate active bone formation areas, which differ from the mature CB of the socket wall. The circled area indicates osteon formation. (B1 and B2) group II (+ ve control) at 2 and 6 weeks, respectively. B1, the extraction socket is filled with dense granulation tissue (stars) and a small area of NB, which contains prominent, large-sized osteocytes (curved arrow) and multiple dense bone resting lines (arrows). It is separated from the CB of the socket wall by a dense, stained line (arrowhead). B2, the socket is filled with dense fibrous granulation tissue (star) and thin bone trabeculae of NB around BM spaces, and many prominent, large-sized osteocytes (curved arrow). The CB contains dense bone resting lines (arrows). The circled area indicates osteon formation. (C1 and C2) group III (Gelatin) for 2 and 6 weeks, respectively. C1, the extraction socket is filled with dense granulation tissue (stars) and a small area of NB separated from the CB of the socket wall, and it also contains prominent, large-sized osteocytes (curved arrow) and multiple dense bone resting lines (arrows). C2, the socket area is filled with dense fibrous granulation tissue (stars) mixed with thick bone trabeculae of NB-1 and NB-2 separated by BM spaces, also contains many osteocytes (curved arrows), and contains densely stained lines (arrows) demarcating its boundary with CB of the socket wall. (D1 and D2) group IV (G–SBG) at 2 and 6 weeks, respectively. D1, the socket area is filled with dense fibrous granulation tissue (star) mixed with a small free mass of bone NB-1 and another NB-2 around CB of the socket wall, both of which contain many osteocytes (curved arrows) and densely stained lines (arrows). D2, the socket is filled with dense fibrous granulation tissue (stars) and thin bone trabeculae of NB, which contains many prominent, large-sized osteocytes (curved arrow), large BM spaces, and mature osteon (circled area). CB of the socket wall also contains large-sized osteocytes (curved arrow). (E1 and E2) group V (G–BBG) at 2 and 6 weeks, respectively. E1, the socket is filled with dense fibrous granulation tissue (stars), separating the native CB of the socket wall from the thick bone trabeculae of NB, which contains many large osteocytes (curved arrow), bone resting lines (arrow), and many BM spaces. The circled areas indicate osteon formation. E2, the NB bone matrix filling the extraction socket contains many dense lines (arrow), large osteocytes (curved arrow), and vascular BM spaces, which differ from the mature CB of the socket wall. The circled areas indicate osteon formation. The densely stained line (arrowhead) lines the NB from the native CB of the socket wall. (Masson’s trichrome stain X 400)

*Group I (-ve control):* After 2 weeks, it showed dense fibrous granulation tissue that filled the socket area, mixed with the newly formed bone, containing dense bone resting lines. Then, at six weeks, it depicted newly formed bone matrix filling the extraction socket with osteon formation, containing many large osteocytes, numerous vascular bone marrow spaces, and multiple dense bone-resting lines, indicating active bone-forming areas distinct from the mature CB of the socket wall (Fig. 6-A1 and A2).

*Group II (*+ *ve control):* After two weeks, the extraction socket appeared filled with dense granulation tissue and a small area of NB, which contained prominent, large-sized osteocytes and multiple dense bone resting lines. It appeared separated from the CB of the socket wall by a dense, stained line. After six weeks, the socket was filled with dense fibrous granulation tissue, thin bone trabeculae of NB around BM spaces, and many prominent, large osteocytes. Osteon formation was also detected during this period (Fig. 6-B1 and B2).

*Group III (Gelatin):* After two weeks, the extraction socket appeared filled with dense granulation tissue and a small area of NB separated from the CB of the socket wall, and it also contained prominent, large-sized osteocytes and multiple dense bone resting lines. After six weeks, the socket area was filled with dense fibrous granulation tissue, mixed with thick bone trabeculae separated by BM spaces, and containing many osteocytes. It also contained densely stained lines demarcating its boundary with the CB of the socket wall (Fig. 6-C1 and C2).

*Group IV (G–SBG):* After two weeks, the socket area appeared filled with dense fibrous granulation tissue mixed with a small free mass of new bone around the CB of the socket wall, which contained many osteocytes and densely stained lines. After six weeks, the socket was filled with dense fibrous granulation tissue and thin bone trabeculae of NB, which had many prominent, large-sized osteocytes, large BM spaces, and a mature osteon. Also, the CB of the socket wall contained large-sized osteocytes (Fig. 6-D1 and D2).

*Group V (G–BBG):* After two weeks, the socket was filled with dense fibrous granulation tissue, separating the native CB of the socket wall from the thick bone trabeculae of NB, which contained many large osteocytes, bone resting lines, many BM spaces, and a mature osteon. After six weeks, the NB bone matrix filling the extraction socket contained many dense lines, mature osteons, large osteocytes, and vascular BM spaces, which differed from the mature CB of the socket wall (Fig. 6-E1 and E2).

### Histomorphometric analysis

**Fig. 7:**
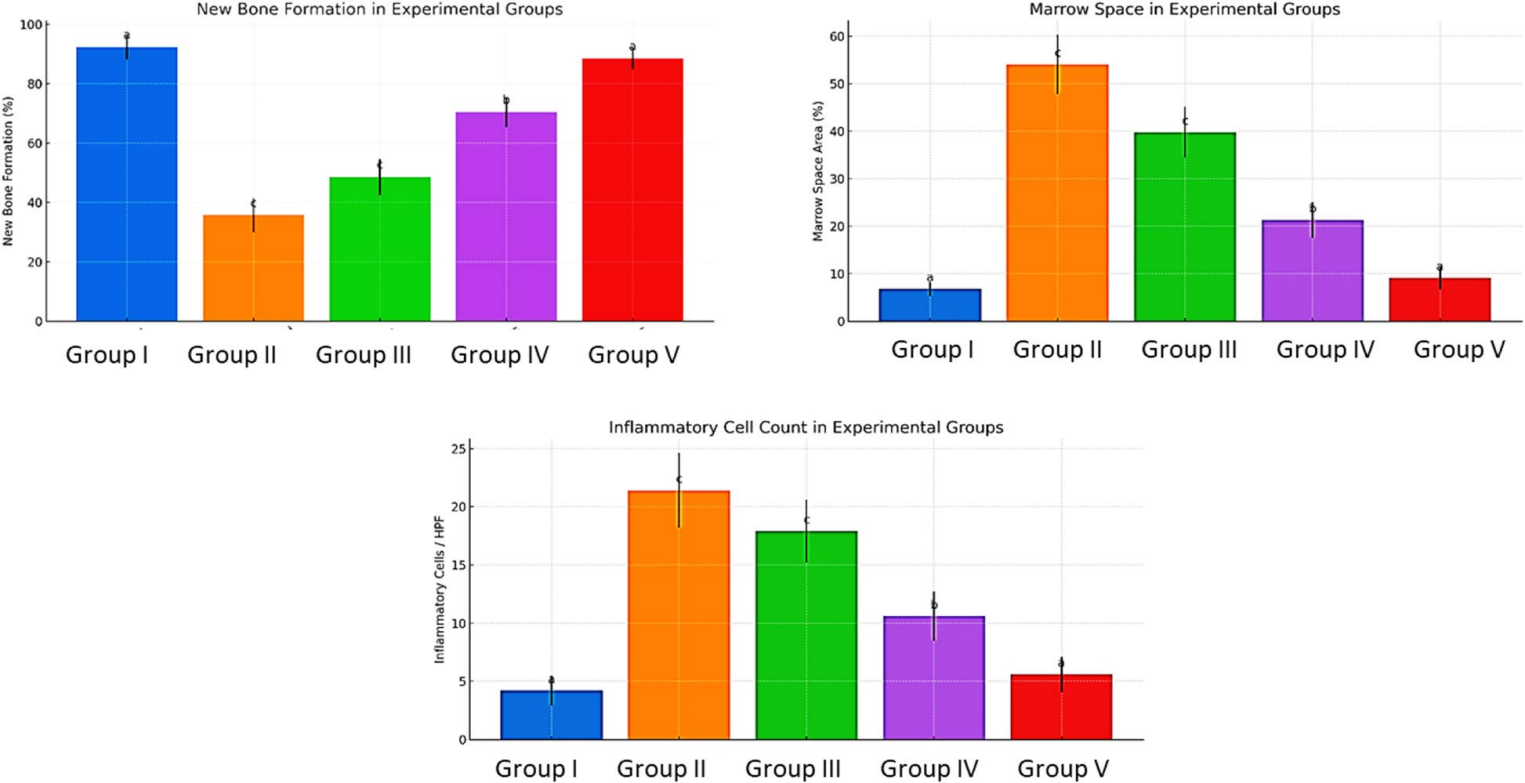
Bar charts illustrate (**a**) percentage of new bone formation, (**b**) marrow space area (%), and (**c**) inflammatory cell count (cells/HPF) in extraction sockets across experimental groups at *2 weeks*. Data are presented as mean ± SD. Different lowercase letters above the bars indicate statistically significant differences between groups (*p* < 0.05), as determined by one-way ANOVA followed by Tukey’s post hoc test. Data are presented as mean ± standard deviation (mean ± SD), *n* = 6

*At 2 weeks,* quantitative histomorphometric analysis revealed significant differences between the experimental groups in new bone formation, marrow space, and inflammatory cell count (Fig. 7).

Regarding the new bone formation (%) (Fig. 6a), Groups I and V showed the highest values (approximately 55% and 52%, respectively), with no significant difference between them (*p* > 0.05). In contrast, Group II showed the lowest value (16%), which was significantly lower than all other groups (*p* < 0.05). Group III demonstrated a moderate increase (28%), while Group IV showed a further significant increase (43%) compared to Groups II and III (*p* < 0.05).

Regarding the marrow space (%) (Fig. 6b), Group II showed the highest percentage (69%), which was significantly higher than that of all other groups (*p* < 0.05). Group III showed an intermediate value (57%), followed by Groups IV and V (45% and 41%, respectively), which were not significantly different from each other. Group I exhibited the lowest marrow space (38%), which was significantly lower than in Groups II and III (*p* < 0.05).

For inflammatory cell count (cells/HPF) (Fig. 6c), Group II showed the highest level of inflammation (26 cells/HPF), significantly greater than all other groups (*p* < 0.05). Group III followed (22 cells/HPF), then Group IV (15 cells/HPF). Groups I and V exhibited the lowest inflammatory cell counts (7 and 9 cells/HPF, respectively), with no significant difference between them (*p* > 0.05).

Collectively, at week 2, Groups I (-ve control) and V (G–BBG) demonstrated the most favorable outcomes, characterized by enhanced bone formation and reduced inflammation. In contrast, Group II consistently showed the least favorable profile across all parameters.

**Fig. 8:**
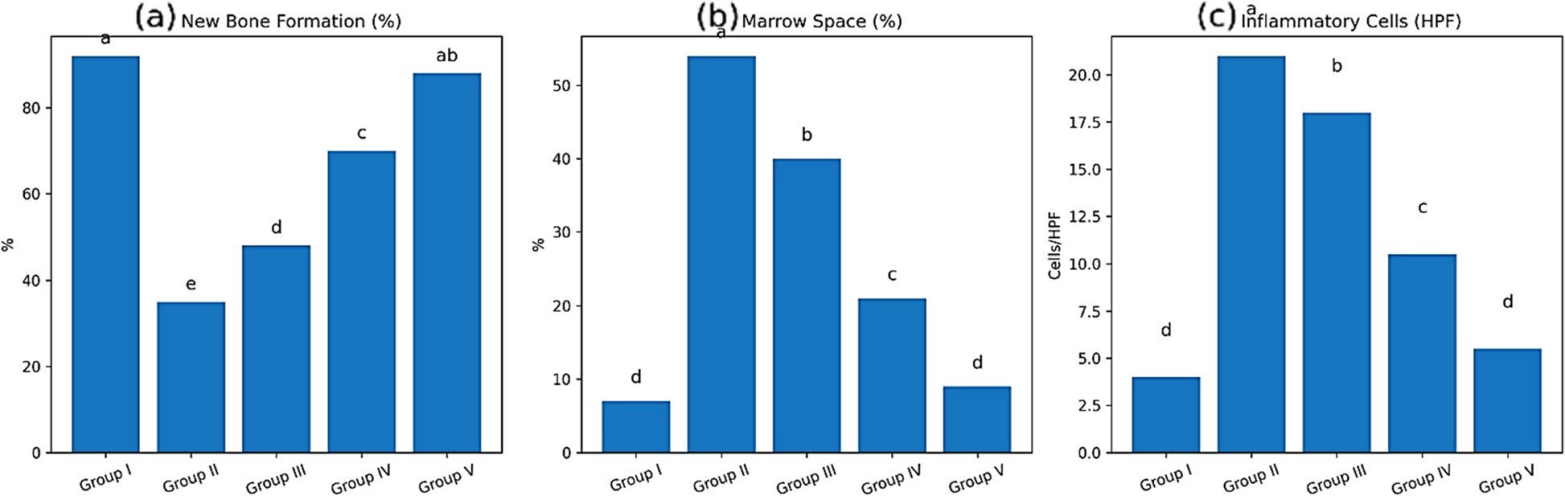
Bar charts illustrate **a** percentage of new bone formation, **b** marrow space area (%), and **c** inflammatory cell count (cells/HPF) in extraction sockets across the experimental groups at *6 weeks*. Data are presented as mean ± SD. Different lowercase letters above the bars indicate statistically significant differences between groups (*p* < 0.05), as determined by one-way ANOVA followed by Tukey’s post hoc test. Data are presented as mean ± standard deviation (mean ± SD), *n* = 6

*At 6 weeks,* quantitative histomorphometric analysis revealed significant differences between the experimental groups in new bone formation, marrow space, and inflammatory cell count (Fig. 8).

For new bone formation (%) (Fig. 7a), Group I exhibited the highest value (92%, a), followed by Group V (88%), which was not significantly different from Group I (ab). Group IV showed a moderate value (70%, c), while Group III demonstrated a lower level (48%, d). Group II presented the lowest bone formation (35%, e), which was significantly lower than all other groups (*p* < 0.05).

Regarding marrow space (%) (Fig. 7b), Group II showed the highest percentage (55%, a), significantly greater than those of all other groups (*p* < 0.05). Group III showed a higher value (40%, b), while Group IV showed a lower value (21%, c). Groups I and V exhibited the lowest marrow space (7% and 9%, respectively) (d for both), with no significant difference between them.

For inflammatory cell count (cells/HPF) (Fig. 7c), Group II demonstrated the highest inflammatory response (21 cells/HPF, a), followed by Group III (18 cells/HPF, b). Group IV showed a moderate level (11 cells/HPF, c). Groups I and V exhibited the lowest inflammatory cell counts (4 and 6 cells/HPF, respectively), (d for both) with no significant difference between them.

Overall, at week 6, Groups I (-ve control) and V (G–BBG) demonstrated the most favorable outcomes, characterized by enhanced bone formation and minimal inflammation. In contrast, Group II consistently showed the least favorable profile across all parameters.

## Discussion

This study investigated the effectiveness of gelatin-based hybrid composites incorporating silicate or borosilicate bioactive glass in enhancing alveolar socket healing in a diabetic rat model. Although silicate bioactive glasses are widely used as osteoconductive materials, their effectiveness in diabetic conditions is limited. Diabetic bones are characterized by chronic inflammation and poor blood supply, and conventional silicate glasses do not directly address these pathological features. In addition, borosilicate glass was selected in this study because it releases boron ions, which have been reported to exhibit anti-inflammatory and pro-angiogenic effects. These biological actions are particularly beneficial in diabetic bone healing, where controlling inflammation and promoting new blood vessel formation are critical for successful regeneration. The results confirmed that the combination of gelatin with BBG significantly enhanced bone regeneration, achieving virtually complete socket healing comparable to that of healthy controls. The findings substantiate the notion that incorporating boron into bioactive glass enhances osteogenesis and alleviates the inadequate bone repair frequently observed in DM.

Selecting the 2-week and 6-week intervals in rodent models in this study was to distinguish between the early cellular response and the late remodeling phase, as in a previous study [30]. At the early Phase (2 Weeks), the histological assessment at this stage is critical for observing angiogenesis, cell recruitment, and initial osteoblastic rimming [31]. In addition, at the Late Phase (6 Weeks), this time point is used to evaluate mature bone formation and the quality of the collagen matrix [32]. Although the 2- and 6-week time points are standardized in bone regeneration research, the biological progression observed in our rodent extraction socket model differs from that in more common models, such as the calvarial critical-size defect [33, 34]. In our study, the 2-week interval revealed a rapid transition from a dense fibrous granulation tissue to an organized osteoid matrix with mature bony area, osteon, particularly in Group V. This is consistent with the high metabolic rate and rich vascularity of the alveolar process in albino rats, which often facilitates faster healing than the calvarial bone. As confirmed by the histological and histomorphometric findings, Figs. 5, 6, 7, and 8

**Scheme 1:**
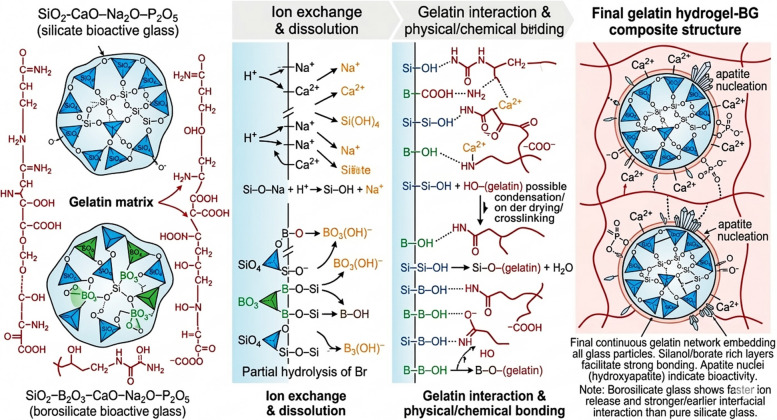
Mechanism between silicate or borosilicate bioactive glass and gelatin hydrogel. This image was drawn using Nano banana AI

In the silicate and borosilicate bioactive glass/gelatin composites, surface hydration of the glass generates silanol and borate hydroxyl groups, along with the release of Ca2 + and other network-modifier ions. These species interact with gelatin carboxylate, amine, and hydroxyl groups via ionic complexation and hydrogen bonding at the glass–polymer interface, forming a physically and, in some cases, chemically coupled hybrid network. The partial substitution of silicate by borate units increases glass reactivity and ion-release rate, thereby enhancing interfacial bonding, water uptake, and controlled degradation of the gelatin matrix, while providing additional hydroxyapatite nucleation for cell response (Scheme 1).

DM is characterized by persistent hyperglycemia and microvascular impairment, which collectively impede angiogenesis, collagen synthesis, and osteoblastic function, leading to prolonged wound and bone healing [35–37]. The compromised blood supply impedes the delivery of oxygen and nutrients to the extraction socket, hence hindering the recruitment of osteoprogenitor cells and the synthesis of new matrix [38, 39]. The restricted bone fill and persistent inflammation observed in the untreated diabetic sockets (Group II) (Fig. 5B1 and B2), and gelatin group (Group III) (Fig. 5C1 and C2) align with these pathophysiological constraints, suggesting that passive scaffolds alone are unable to address diabetic dysfunction [40].

The integration of bioactive glass particles into the gelatin matrix yielded a dynamic, ion-releasing scaffold that stimulates cellular and molecular pathways crucial to bone repair. The G-SBG enhanced healing relative to controls, as evidenced by increased bony bridging and larger bone marrow gaps (Fig. 5D1 and D2). SBG is known to release silicon, calcium, and phosphate ions, which facilitate osteoblast proliferation, collagen synthesis, and angiogenic signaling. This accelerates osseous regeneration [41, 42]. Nevertheless, the extent of bone maturation in G–SBG was inferior to that observed in G–BBG, suggesting that while silicate glass promotes initial bone formation, its regenerative signaling is compromised in DM.

The G-BBG in group V demonstrated the most pronounced osteogenic effect, as indicated by the new bone formation, wide vascular marrow spaces, osteon generation, and absence of inflammatory infiltration, as proven in Fig. 5E1 and E2. The enhanced performance can be attributed to the incorporation of boron ions, which have been shown to accelerate the production of hydroxycarbonate apatite (HCA), promote osteoblast proliferation, and enhance angiogenesis [41, 43–45]. Boron alters the functionality of critical signaling molecules, such as bone morphogenetic proteins (BMPs) and vascular endothelial growth factor (VEGF). Both are essential for linking osseous development with vascular proliferation [46]. Boron has anti-inflammatory properties, as it reduces levels of pro-inflammatory cytokines. This enhances the tissue microenvironment for bone repair [47]. The bioactive glass component operates by a recognized mechanism. Upon contact with biological fluids, the glass network undergoes partial dissolution, liberating Ca^2^⁺, Si^4^⁺, and B^3^⁺ ions that induce supersaturation and facilitate the development of a bone-like hydroxycarbonate apatite (HCA) layer. This layer of osteoblast adhesion and proliferation [48]. Additionally, this layer inhibits osteoclastic activity [49]. The present study revealed the intimate incorporation of residual G–BBG particles into mineralized bone, confirming the composite scaffold's enhanced bioactivity and osteoconductivity.

The incorporation of boron enhances bioactivity; nevertheless, excessive boron may be detrimental due to its potential to elevate pH, osmolarity, and cytotoxicity [50]. The gelatin matrix employed here markedly diminished this risk by regulating ion mobility and the rate of their degradation. The polymeric network maintained the stability of the BBG particles, facilitating the controlled release of ions that sustained osteogenic stimulation without inducing inflammation or necrosis [51]. The reduced swelling ratio seen in the composites, as evidenced in Fig. 4, further corroborates this stabilization effect, ensuring the structure remains robust throughout the healing process.

The present findings support previous research, demonstrating that boron-doped bioactive glasses enhance bone healing and vascularization more efficiently than silicate-based alternatives [52]. The nearly complete healing of the socket observed in the G–BBG group illustrates the synergistic function of the organic (gelatin) and inorganic (borosilicate glass) components in creating a biomimetic scaffold that replicates the architecture and biochemistry of authentic bone tissue. An essential element of this work is the use of a diabetic model that accurately simulates clinical issues related to compromised bone regeneration. The restoration of bone architecture and the resolution of inflammation in the G–BBG group highlight its translational potential to mitigate post-extraction complications in diabetic patients, a population characterized by delayed and insufficient socket healing.

At week 2, Groups I and V showed the highest values of new bone formation (%) (55% and 52%, respectively; Fig. 6a), with no significant difference between them (*p* > 0.05). Also, group V (41%) did not differ significantly from Group I, which exhibited the lowest marrow space (38%). At week 6, for new bone formation (%) (Fig. 7a), Group I exhibited the highest value (92%, a), followed by Group V (88%), which was not significantly different. Groups I and V exhibited the lowest marrow space (7% and 9%, respectively) (Fig. 7b), with no significant difference between them. DM is usually associated with delayed socket healing, reduced mineral density, and increased marrow space volume [53]. The application of the G–BBG composite appeared to stabilize these constraints, restoring the osteogenic potential and maintaining the architectural integrity in Group V to levels comparable to those in healthy physiological conditions in Group I. The released boron has been shown to improve the mineralization of the extracellular matrix and improve the mechanical properties of regenerating bone [54]. Moreover, the released Silicon ions have been shown to upregulate BMP and VEGF expression, stimulating both vascular angiogenesis and osteogenesis [46]. Also, gelatin, a collagen derivative, acts as a biomimetic scaffold, providing an ideal RGD (Arg-Gly-Asp) peptide sequence that promotes cell adhesion and migration, facilitating the infiltration of osteoprogenitor cells [55]. Notably, Macrophage polarization plays a fundamental role in regulating the bone-healing microenvironment. Classically activated M1 macrophages, characterized by the expression of pro-inflammatory cytokines such as IL-1β, are primarily involved in the early inflammatory phase, whereas alternatively activated M2 macrophages, marked by Arginase-1 expression, contribute to inflammation resolution and tissue regeneration [56, 57]. Also, a severely regulated transition from the M1 to M2 phenotype is essential for successful bone repair and remodeling [58]. Moreover, in previous studies, some biomaterials had been reported to modulate the immune response by favoring M2 polarization, thereby creating a pro-regenerative microenvironment conducive to bone formation [59]. Nevertheless, further mechanistic investigations, including cytokine profiling and gene expression analyses, are warranted to elucidate the immunomodulatory pathways involved in these groups fully.

Our findings revealed enhanced bone formation and reduced inflammation in Groups I and V, as shown in Fig. 5 A1,2 and E1,2, respectively, suggesting a favorable microenvironment for tissue regeneration. This effect may be attributed to the modulation of key molecular pathways involved in osteogenesis and inflammation. It is well established that bone remodeling is regulated by the balance between receptor activator of nuclear factor kappa-B ligand (RANKL) and osteoprotegerin (OPG). A reduction in inflammation may suppress RANKL expression while promoting OPG activity, thereby inhibiting osteoclastogenesis and enhancing bone formation, as reported in our previous research [60]. Moreover, the observed decrease in inflammatory cell infiltration may indicate downregulation of pro-inflammatory cytokines such as tumor necrosis factor-alpha (TNF-α) and interleukin-6 (IL-6), which are known to impair osteoblast differentiation and promote bone resorption [61, 62]. In contrast, improved bone formation suggests enhanced osteoblastic activity, potentially mediated through pathways such as Wnt/β-catenin signaling, which plays a critical role in bone regeneration [60, 61]. Although the current study did not directly investigate these molecular mechanisms, the histological and quantitative findings provide indirect evidence supporting their involvement.

Notably, the immunomodulatory effect, which ultimately facilitates osteogenesis, is closely associated with enhanced collagen deposition and matrix organization, as demonstrated by histological analyses such as Masson’s trichrome staining [62]. Masson’s trichrome in the G-BBG of group V (Fig. 6 E1 and E2) revealed that at week two, the socket was filled with dense fibrous granulation tissue, separating the native bone of the socket wall from the thick bone trabeculae of NB, which contained many large osteocytes, bone resting lines, many BM spaces, and a mature osteon. After six weeks, the NB bone matrix filling the extraction socket contained many dense lines, mature osteons, large osteocytes, and vascular BM spaces, which differed from the mature CB of the socket wall. Importantly, the findings of this study extend beyond previous reports of wound healing studies by linking the anti-inflammatory effect of boron directly to histological evidence of matrix formation using Masson’s stains [63].

## Conclusion

This research shows that incorporating bioactive glass particles into a gelatin matrix significantly accelerates the healing of alveolar sockets in diabetic rats. G–BBG exhibited superior bioactivity and regenerative efficiency compared to G–SBG, generating new bone comparable to that of healthy, non-diabetic individuals. The results highlight the potential of G–BBG hybrid composites as biomimetic scaffolds for clinical applications to preserve the alveolar ridge and regenerate bone tissue, particularly in patients with compromised healing capacity, such as those with DM. Future research should focus on the extended evaluation of mechanical stability, biodegradation, and the molecular mechanisms that promote the osteogenic attributes of boron-infused glass composites.

### Recommendation

Although the profits of the proposed G–BBG composite in this study are promising, it is crucial to evaluate its efficacy in larger animal models to assess its therapeutic potential better.

### Study limitations

The present study relied primarily on histological and histomorphometric assessments; future investigations should incorporate molecular and immunohistochemical examinations to elucidate the specific signaling pathways involved. Also, this study lacks in vitro investigations, including assays of cell viability, osteogenic differentiation, and cytokine profiling, to further elucidate the underlying mechanisms of bone regeneration. Additionally, long-term studies are needed to evaluate complete remodeling, mechanical strength, and biodegradation behavior of the composite scaffold in vivo.

## Data Availability

The datasets generated and analyzed during the current study are available from the corresponding author upon reasonable request.

## Abbreviations

BG: Bioactive glass
SBG: Silicate bioactive glass
BBG: Borosilicate bioactive glass
G-SBG: Gelatin/silicate bioactive glass composite
G-BBG: Gelatin/borosilicate bioactive glass composite
DM: Diabetes mellitus
H&E: Hematoxylin and eosin
HCA: Hydroxy-carbonate apatite
SEM: Scanning electron microscopy
